# Production of *Aspergillus niger* biomass on sugarcane distillery wastewater: physiological aspects and potential for biodiesel production

**DOI:** 10.1186/s40694-018-0045-6

**Published:** 2018-01-16

**Authors:** Graziella Chuppa-Tostain, Julien Hoarau, Marie Watson, Laetitia Adelard, Alain Shum Cheong Sing, Yanis Caro, Isabelle Grondin, Isabelle Bourven, Jean-Marie Francois, Elisabeth Girbal-Neuhauser, Thomas Petit

**Affiliations:** 1 Antenne sud du laboratoire de chimie des Substances Naturelles et des Sciences des Aliments (LCSNSA), EA 2212, Université de la Réunion, UFR des Sciences et Technologies, 15 Avenue René Cassin, CS 92003, 97744 Saint-Denis Cedex 9, France; 2 Laboratoire de Physique et Ingénierie Mathématique pour l’Energie et l’Environnement (PIMENT), EA 4518, Université de la Réunion, UFR Sciences de l’Homme et de l’Environnement, 117 rue Général Ailleret, 97430 Le Tampon, France; 3 Groupement de Recherche Eau Sol Environnement (GRESE), EA 4330, Université de Limoges, Faculté des Sciences et Techniques, 123 Avenue A. Thomas, 87060 Limoges Cedex, France; 4 LISBP, UMR INSA-CNRS &/INRA 792, 135 Avenue de Rangueil, 31077 Toulouse Cedex 4, France; 5 Laboratoire de Biotechnologies Agroalimentaire et Environnementale (LBAE), EA 4565, Université de Toulouse III, Institut Universitaire de Technologie, 24 Rue d’Embaquès, 32000 Auch, France; 6 Present Address: Département Hygiène Sécurité Environnement (HSE), Institut Universitaire de Technologie, Université de La Réunion, 40 Avenue de Soweto, 97410 Saint-Pierre, France

**Keywords:** Sugarcane distillery wastewater, Vinasse, Distillery spent wash, *Aspergillus niger*, Biomass production, Bioremediation, Biodiesel, Lipids

## Abstract

**Background:**

Sugarcane distillery waste water (SDW) or vinasse is the residual liquid waste generated during sugarcane molasses fermentation and alcohol distillation. Worldwide, this effluent is responsible for serious environmental issues. In Reunion Island, between 100 and 200 thousand tons of SDW are produced each year by the three local distilleries. In this study, the potential of *Aspergillus niger* to reduce the pollution load of SDW and to produce interesting metabolites has been investigated.

**Results:**

The fungal biomass yield was 35 g L^−1^ corresponding to a yield of 0.47 g of biomass/g of vinasse without nutrient complementation. Analysis of sugar consumption indicated that mono-carbohydrates were initially released from residual polysaccharides and then gradually consumed until complete exhaustion. The high biomass yield likely arises from polysaccharides that are hydrolysed prior to be assimilated as monosaccharides and from organic acids and other complex compounds that provided additional C-sources for growth. Comparison of the size exclusion chromatography profiles of raw and pre-treated vinasse confirmed the conversion of humic- and/or phenolic-like molecules into protein-like metabolites. As a consequence, chemical oxygen demand of vinasse decreased by 53%. Interestingly, analysis of intracellular lipids of the biomass revealed high content in oleic acid and physical properties relevant for biodiesel application.

**Conclusions:**

The soft-rot fungus *A. niger* demonstrated a great ability to grow on vinasse and to degrade this complex and hostile medium. The high biomass production is accompanied by a utilization of carbon sources like residual carbohydrates, organic acids and more complex molecules such as melanoidins. We also showed that intracellular lipids from fungal biomass can efficiently be exploited into biodiesel.

**Electronic supplementary material:**

The online version of this article (10.1186/s40694-018-0045-6) contains supplementary material, which is available to authorized users.

## Background

Sugarcane molasses fermentation and distillation into rum lead to the production of wastewater called stillage, vinasse, distillery wastewater or distillery spent wash. Every produced litre of ethanol brings about from 10 to 18 litres of sugarcane distillery wastewater (SDW) depending on distillation process and waste treatment [1]. SDW is a dark brown effluent characterized by a specific obnoxious odour, a high chemical oxygen demand (COD) and a total organic carbon (TOC) that can reach up to 120 and 17 g L^−1^ respectively [2, 3]. According to Wilkie et al. [4], COD is 4–5 times higher in sugarcane molasse stillage as compared to sugarcane juice stillage. Depending on the sugarcane origin and the industrial process for ethanol production, intrinsic composition of SDW can vary significantly. They generally have acidic pH (from 3.8 to 5) due to the presence of organic acids produced by the yeasts during the alcoholic fermentation process [5]. A high mineral load was also reported due to the presence of sulphur, potassium, phosphate, calcium and sodium [6, 7]. The high organic load of SDW is mainly composed of melanoidins which are produced through Maillard reactions between sugars and proteins and caramels from overheated sugars that are responsible for their colour and odour. Vinasse also contains other refractory materials such as phenolic compounds, anthocyanins, tannins and furfurans (for example hydroxyl methyl furfural) which can reach up to 10 g L^−1^ [8–10]. The colloidal nature of caramels makes them resistant to decomposition and toxic to microflora [11]. SDW also contains residual sugars and soluble proteins generated by the fermenting yeasts [12].

All these characteristics combined with the high volume of SDW produced worldwide cause significant environmental issues. Over the last decades and due to their high inorganic loads, SDW have been widely used as agricultural fertilizer [13] but spreading is made now statutory difficult due to their low pH, dark colour and chemical content which may be responsible for groundwater contamination and soil compaction [14]. Their high polluting loads lead to a modification of the soil composition and can cause eutrophication of the waterways because of the presence of proteins residues and furfurals [4, 15]. Moreover, melanoidins cause reduction of sunlight penetration, of photosynthetic activity and of dissolved oxygen concentration in natural aqueous environment, whereas on land, they cause reduction of soil alkalinity and inhibition of seed germination. In consequence, phenolic compounds and melanoidins may inhibit the activity of microorganisms contained in soils and aquatic environments [9, 10, 15].

Several methods have been described in literature for the use and disposal of SDW [10, 15, 16]. Among them, aerobic treatment of SDW has been proposed for decolourisation and COD reduction purposes. A number of microorganisms, such as yeast and fungi were found to be able to degrade melanoidins and to significantly decrease the COD vinasse [10]. Preliminary experiments performed in the lab (unpublished data) showed that only a few molds are capable of growing on crude SDW, such as *Aspergillus* strains and anamorphs. *Aspergillus niger* is able to grow on a large variety of substrates, a wide range of temperatures (6–47 °C) and pH (1.4–9.8), explaining the ubiquitous occurrence of this species that is encountered with a higher frequency in warm and humid environments [17]. *A. niger* is also known to be a good producer of extracellular enzymes with significant industrial importance, including amylases, proteases, pectinases, lipases as well as valuable molecules with industrial interest such as citric, oxalic or gluconic acids [18, 19]. *A. niger* is also used for organic waste enhancement [20] and its capacity to grow on diluted or supplemented SDW was observed [21–23]. However, the physiological growth characteristics of this micro-organism cultured in crude sugarcane distillery spent wash has not yet been reported. In addition, bioremediation and potential valorisation of crude SDW were estimated through the production of *A. niger* biomass as a valuable source for biofuel.

## Methods

### Fungal strains, growth conditions and culture media

The strain used in this study was *Aspergillus niger* MUCL 28820 from BCCM (Brussels, Belgium) strain collection. The strain was maintained routinely on potato dextrose agar plates (PDA). A suspension of *A. niger* spores was prepared as follow: spores, grown on PDA and incubated at 28 °C for 72 h, were harvested using a glass loop and suspended in sterile physiological water (NaCl 0.8%). Cellular concentration was calculated using a Thoma-Zeiss counting chamber. Growth experiments were performed during 10 days, after inoculation with 100 µL of spore suspension. Ten flasks containing 50 mL of sterile SDW liquid medium at a starting concentration of 10^5^ spores mL^−1^ were plugged with sterile cotton carded and placed on a rotary shaker at 150 rpm at 28 °C. Assays were performed in three independent biological experiments. Every day, at the same hour, the biomass of three flasks was harvested by filtration for further analysis (see below) and this was repeated until day 10.

SDW medium was prepared as follows: raw SDW (85 °C) was harvested in decontaminated barrel directly from the output of the distillation column from distillery “Rivière du Mât” (Saint-Benoit, Reunion Island) and cooled to room temperature. SDW from the distillery still contains the residual inactivated yeast biomass used during alcohol fermentation. SDW samples were harvested during the sugar production period (i.e. between July and December) in 2012 and in 2014 and were frozen and stored at − 20 °C until use. For each experiment, a new batch of frozen DSW was thawed and then sterilized by autoclaving for 20 min at 121 °C. Such autoclaved DSW medium was microbiologically stable over time (Additional file 1).

**Fig. 1:**
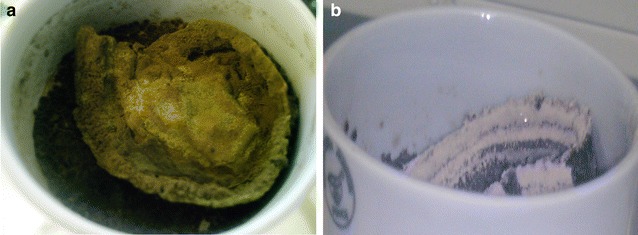
Photographs of fungal biomass (**a**,** b**) produced during growth on SDW (day 10). Filtrated *A. niger* cell pellets were harvested under vacuum on Whatman No. 1 paper using Büchner funnel (see “Methods” section)

After filtration of 50 mL of SDW (through a cellulose filter paper Whatman No. 1—porosity 11 µm), the filtrates and filters containing the total suspended solids (see example on Fig. 1) were both dried during 24 h in an oven at 105 °C. The obtained dried masses were reported to 50 mL allowing to determinate the corresponding concentrations in total dissolved solids (TDS) and total suspended solids (TSS), respectively. Mineral matters present in the SDW filtrates were measured according to Analytical Procedure of National Renewable Energy Laboratory by incineration of 10 mL of SDW filtrates at 550 °C for 3 h [24]. The pH of the SDW filtrate was measured using a pH-meter Denver Instrument. Soluble COD and Total Nitrogen (TN) determinations were carried out on the SDW filtrate using a DR 2800 spectrophotometer (Hach Lange, Dusseldorf) and the appropriate analytical kits [25]. Samples were adequately diluted with sterile deionized water and analysed according to manufacturer’s instructions. SDW filtrates were diluted to 1/100 and their optical density was measured at 475 nm using a spectrophotometer Genesys 10 UV Deionised water was used as blank.

*Lipid accumulation medium (LAM)* contained 30 g L^−1^ glucose, 1.5 g L^−1^ yeast extract, 0.5 g L^−1^ NH_4_Cl, 5.0 g L^−1^ Na_2_HPO_4_ (12H_2_O), 7.0 g L^−1^ KH_2_PO_4_, 1.5 g L^−1^ MgSO_4_ (7H_2_O), 0.1 g L^−1^ CaCl_2_ (2H_2_O), 0.01 g L^−1^ ZnSO_4_ (7H_2_O), 0.08 g L^−1^ FeCl_3_ (6H_2_O), 0.1 mg L^−1^ CuSO_4_ (5H_2_O), 0.1 mg L^−1^ Co(NO_3_)_2_ (6H_2_O), 0.1 mg L^−1^ MnSO_4_ (5H_2_O) and pH was adjusted to 5.5 according to [26].

### Fungal biomass determination

The concentration of total suspended solids (TSS) in the broth medium of each culture flask was determined by filtration of 50 mL of SDW (treated or not b*y A. niger*) through a cellulose filter paper Whatman No. 1 (porosity 11 µm) previously dried for 24 h at 105 °C. The insoluble suspended solids kept on the filter (see example on Fig. 1) were dried during 24 h in an oven at 105 °C and the obtained dry mass was weighed to provide TSS concentration. Therefore, TSS contained the fungal biomass produced during growth of *A. niger* as well as the initial suspended yeast biomass contained in raw SDW. Fungal biomass was thus estimated by subtracting the total suspended matter of raw SDW to the total mass harvested on the filter.

### Analytical methods

#### Determination of carbohydrates and organic acids from filtrates of crude and pre-treated SDW

The carbohydrate concentration of the filtrates collected from crude SDW and SDW treated with *A. niger,* were analysed by High-Pressure Liquid Chromatography (HPLC) (Dionex Ultimate 3000) using an Evaporative Light Scattering (ELS) detector (VARIAN) and a Hi-plex Ca column (Varian, C18 bound—7.7 mm of diameter × 300 mm of length). A mobile phase of ultrapure water with a flow of 0.4 mL min^−1^ was used. The oven temperature was programmed at 80 °C. Alternatively, High-Pressure Ion Chromatography (HPIC) (Dionex) using a pulsed amperometric detector and a CarboPack PA1 column was used. A mobile phase of NaOH (150 mM) at 1.5 mL min^−1^ was used at an oven temperature of 30 °C. Analysis of organic acids was also performed by HPLC (Dionex Ultimate 3000), using a UV detector at 214 nm and an OA Acclaim column (Varian, Silica, C18 bound, reverse phase, 4.6 mm of diameter × 150 mm of length). The mobile phase was composed of 100 mM Na_2_SO_4_ set at pH of 2.65 with methanesulfonic acid (Sigma-Aldrich, CAS number 75-75-2) and the flow rate was 0.6 mL min^−1^. For all analyses, 20 μL of samples diluted 100-fold for organic acids and 50-fold for carbohydrates in water were injected using an automatic autosampler. The identification and the quantification of carbohydrates (mannitol, glucose, fructose, sucrose) and organic acids (itaconic acid, trans-aconitic acid, citric acid, isocitric acid, oxalic acid) were made by determination of retention time of the commercial standards and establishment of calibration curves using external standard method. Treatment of the results was done using *Chromeleon* 7.2 Chromatography Data System (Dionex).

#### SEC profiles obtained from filtrates of crude and pre-treated SDW

A 5-days fermented SDW was chosen for this experiment because at this stage of the growth (mid-exponential phase), most of the sugars and organic acids remains unchanged while biomass already reached more than 10 g L^−1^ DW, suggesting that others classes of molecules were used preponderantly for growth of the cells. The filtrates of crude SDW and 5-days treated SDW with *A. niger* were ten times diluted with phosphate buffer (pH 7.0) and filtered (through a 0.45 µm filter) before injection in a HPLC system Äkta-Purifier (GE Healthcare). As previously described by [27], two SEC columns were connected in series in order to obtain a wide resolving range: the Superdex peptide 10/300 GL column with a resolving range from 0.1 to 7 kDa was placed before the second Superdex 200 10/300 column (GE Healthcare) with a resolving range from 10 to 600 kDa. A similar volume of 0.1 ml of the two samples previously diluted in PBS was injected and elution of the molecules was performed at room temperature using a 50 mM potassium phosphate buffer (pH 7.0) as the mobile phase at a flow-rate of 0.4 mL min^−1^ and fractions of 2 mL each were collected. The Unicorn 5.1 software (GE Healthcare) delivered on the Akta purifier allows to either multiply or divide the chromatograms with a constant factor: depending on the total COD concentration of the sample, the obtained chromatogram can be thus standardized per mg of COD. Peak area integration of the standardized chromatograms was performed by the Unicorn 5.1 software. The SEC columns were calibrated for molecular weight determination using a mixture of standard proteins of known molecular weight between 12 and 669 kDa (HMW and LMW calibration kits, GE Healthcare). Calibration showed a linear relationship between the log of molecular weight (MW) and the elution volume (Ve) of the standards according to the following equation:

$${\text{Log}}\,\left( {\text{MW}} \right) = - 0.1536{\text{ Ve }} + \, 8.5794 \, \left( {\mathbf{1}} \right)\;\, \, \left( {{\text{R}}^{2} = \, 0.9976} \right)$$

with MW expressed in Da and Ve in mL.

#### EEM profiles obtained from filtrates of crude and pre-treated SDW

A three-dimensional excitation emission matrix (3-D EEM) was determined on the SDW filtrates (raw or treated with *A. niger*) and on the SEC fractions, using a spectrofluorophotometer (Shimadzu RF-5301 PC) with a 150-W Xenon lamp as the excitation source. Excitation scans were performed from 220 to 450 nm at 10 nm increments; emission scans were collected from 220 to 500 nm. The fluorescence data was processed using the Panorama Fluorescence 3.1 software (LabCognition, Japan). Prior to measurements, fractions of SEC samples were diluted by 3–100 times using 50 mM phosphate buffer (pH 7.0 ± 0.1) to avoid fluorescence signal saturation. However, due to the impact of water noise, only emissions obtained at excitation wavelengths exceeding 275 nm were considered for a wavelength emission exceeding 375 nm. Gallic acid (Sigma), used as polyphenols standard [28] was also diluted in phosphate buffer for analysis. Fluorescence was measured using a 1.0 cm quartz cell.

### Lipid extraction from *A. niger* biomass and conversion into biodiesel

Intracellular lipids were extracted using a pressurized liquid extraction method (PLE). 200 mg of lyophilized biomass was mixed with Fontainebleau sand to fill a 10 mL stainless steel vial suitable for PLE. The extraction was carried out using chloroform/methanol (2/1) at 100 °C during 10 min (three times), then 10 mL of water was added to the extract and thoroughly mixed. Two phases were obtained after overnight separation. The organic phase was dried over anhydrous MgSO_4_, filtered and concentrated using a rotative evaporator. Finally, the concentrate was suspended in 3 mL CHCl_3_, transferred to a pre-weighed bottle and evaporated overnight. The bottle was weighted to determine the mass of extracted lipids. Transesterification was performed according to a procedure described by [29]. Briefly, 5 mL of 2% H_2_SO_4_/CH_3_OH (v/v) was added to the extracted lipids and the mixture was reflux heated at 70 °C during 1 h under constant stirring. The flasks were then cooled at room temperature. Next, 2 mL of hexane and 0.75 mL of distilled water were added to the flasks and mixed. The two phases were allowed to separate and the upper hexane layer was recovered and dried over anhydrous magnesium sulphate.

Analysis of the fatty acid composition was carried out on a CP3800 Gas chromatograph (Varian) equipped with a SG BPX-70 capillary column (50 m × 0.22 mm × 0.25 µm) and a flame ionization detector. The operating conditions were 240 °C injector temperature, 260 °C detector temperature, 1.3 mL min^−1^ flow rate and oven temperature programmed from 120 to 230 °C at 3 °C min^−1^ then 230 °C for 17 min. 0.5 µL of transesterification product was injected and subjected to a split ratio of 5 at 0.5 min then 50 at 5 min. The percentage of the peak area was assumed to be the percentage content of the corresponding compounds.

## Results and discussion

### Physico-chemical characteristics of SDW from Reunion Island

#### Physico-chemical parameters of raw SDW

To characterize our raw materials, main physico-chemical parameters of the collected SDW samples were assayed. Results are presented in Table 1. pH value of raw SDW (4.6 pH units) was comparable to average pH values (3.8–4.6) reported by [30] for SDW from others countries. Similarly, COD (107 g L^−1^) and TDS (114 g L^−1^) of SDW from Reunion Island were in the same range of order than the one reported for SDW from different origins that ranged from 42 to 121 and from 80 to 100 g L^−1^ respectively, with the outstanding exception of TDS of Brazilian SDW that reached up to 152 g L^−1^ [30–32]. Chemical composition of SDW filtrate showed a TN content of 2.32 g L^−1^ and a total mineral content of 38.5 g L^−1^. The first parameter was globally in good agreement with data of SDW from different southern countries, i.e. 1.23–4.8 g TN L^−1^ whereas the latter was higher than literature data namely 10.7–28.9 g L^−1^ [30, 33]. Overall, these physico-chemical parameters confirmed that SDW from Reunion Island are industrial wastes with high polluting organic and mineral loads that can be responsible for dangerous environmental disorders.

**Table 1:** Comparison of physico-chemical parameters of raw SDW and treated SDW filtrates obtained after 10 days of aerobic fermentation by *A. niger*

Physico-chemical parameters	Laboratory data	
	Raw SDW (day 0)	Fermented SDW (day 10)
pH	4.6	5.4
COD (g L^−1^)	107	50
TDS (g L^−1^)	114 ± 12.8	89 ± 7.07
TSS^a^ (g L^−1^)	8.13 ± 1.41	43.42 ± 1.2
TN (g L^−1^)	2.32	1.7
Ashes (g L^−1^)	38.5 ± 2.33	43.2 ± 1.94
C/N	11.8	11.3
OD_475nm_	34.5	25.2

SDW was incubated aerobically during 10 days with *A. niger* as explained in Methods section

*TDS* total dissolved solids, *TSS* total suspended solids, *COD* chemical oxygen demand, *TN* total nitrogen, *C/N* carbon/nitrogen, *OD*_*475nm*_ optical density measured at 475 nm

^a^Except for TSS that were measured on insoluble suspended solids

#### Physico-chemical parameters of SDW after treatment with *A. niger*

To assess the bioremediation potential of *A. niger*, the physico-chemical parameters of SDW were measured 10 days after the inoculation of the fungal spores in SDW. As shown in Table 1, a pH increase (from 4.6 to 5.4) and a decrease in OD_475nm_ (linked to decolourisation) were observed during aerobic fermentation of SDW. TDS were significantly reduced from 114 to 89 g L^−1^ and this essentially concerns organic matter reduction since the mineral load was not significantly modified. A reduction of COD and TN by 53 and 27% respectively were observed, indicating a significant decrease of the organic pollutant load of SDW. The pH increase could result from the degradation of organic substances with peptidic moieties or with amino group like humic substances, melanoidins, peptides or amino acids initially contained in SDW medium. The carbon/nitrogen (C/N) ratio remained globally unchanged indicating that the fertilizing potential of SDW remained the same after the fermentation process.

Bioremediation potential of *A. niger* on SDW was partially described in literature. A maximal colour elimination of 69% and a maximal COD removal of 75% were obtained when MgSO_4_, KH_2_PO_4_, NH_4_NO_3_ and a carbon source were added to SDW [34]. Also, immobilized *A. niger* resulted in a 80% decolourisation of previously anaerobically biodigested SDW [35]. Finally, the observed COD and colour decrease suggested that refractory molecules like melanoidins and other aromatic compounds were hydrolysed into simple ones. Such hydrolysis of some refractory compounds may contribute to the strong decrease of the measured COD (− 53%) because of the release of acidic moieties impacting on the oxidation degree of the polymers. In this way, qualitative characteristics of organic matter from raw and pre-treated SDW were investigated.

### Physiology of *A. niger* cultured on SDW

#### Global biomass production

Concomitantly to the modification of some physicochemical parameters, important biomass production was observed in the SDW medium after 10 days of *A. niger* aerobic growth (Fig. 1 and Table 1). Fungal growth was evaluated by measurement of the total suspended solids in the broth medium that reached 43.4 g L^−1^ after 10 days. Given that the residual yeast biomass contained in raw SDW amounted to 8.1 g L^−1^, a net production of 35.3 g L^−1^ of fungal biomass in SDW after 10 fermentation days could then be estimated. In addition to carbon containing substrates, residual dead yeasts contained in raw SDW (inactivated during the distillation process and newly sterilised before use) are most likely to play a role during growth such as bringing important nitrogen source. Consequently, SDW was considered as an interesting growth medium for *A. niger* biomass production. In their study, Oshoma et al. [21] demonstrated that the final concentration of *A. niger* biomass could be increased from 1.63 to 2.75 g L^−1^ Dry weight (DW) after nitrogen supplementation of cassava whey by yeast extract (2 g L^−1^). By comparison, growth of *A. niger* on SDW from Brazil distilleries in which the yeast biomass was removed led to a biomass production of 8–13 g L^−1^ DW [22]. Here, the biomass production was much higher since until 35 g L^−1^ of *A. niger* biomass can be produced after 10 days on raw sugarcane vinasse without any supplementation. Considering that total organic matter of raw vinasse corresponds to TDS without ashes (75.5 g L^−1^), a high biomass yield of 0.47 g g^−1^ on initial organic compounds can be reached. This yield is similar to that obtained by [36] that investigate the capability of *A. niger* to utilize lignocellulose-derived compounds after thermochemical pre-treatment of spruce wood chips. However, because of the presence of fermentation inhibitors, the pre hydrolysate medium had to be diluted 2 or 4 times to allow *A. niger* growth with a maximal volumetric biomass yield of 7 g L^−1^ and a biomass yield on initial carbon source of 0.46 g g^−1^.

#### Sugar consumption

**Fig. 2:**
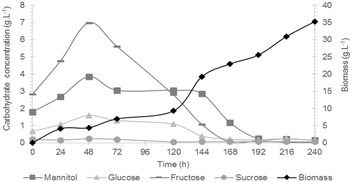
Carbohydrates and mannitol profiles during *A. niger* growth on SDW media during 10 days. mannitol ( ), glucose ( ), fructose ( ), sucrose ( ) and biomass ( )

To explain the physiological behaviour of *A. niger* on raw SDW, carbohydrate content was monitored in the medium during the 10 days of fermentation process (Fig. 2). During the first 48 h, residual concentration of glucose, fructose and mannitol was increased by a factor of 2 and a maximal concentration of 7 g L^−1^ of fructose, 1.6 g L^−1^ of glucose and 4 g L^−1^ of mannitol were measured 2 days after inoculation of the fungal spores in the SDW medium. In the meantime, total fungal biomass increased slightly up to 4.37 g L^−1^. Accumulation of these monosaccharides in the early stage of the exponential growth phase strongly suggested that some complex compounds were readily released by hydrolytic enzymes secreted by *A. niger*. In a second period, from 48 h (day 2) to 192 h (day 8), fructose, glucose and mannitol were gradually consumed, until complete exhaustion that occurred at 168 h (day 7) for glucose and fructose, and at 192 h (day 8) for mannitol. Fungal biomass that increased very weakly in the first period then suddenly increased during the period of sugars assimilation (from day 2 to 8, it increased from 0.8 to 25 g L^−1^) and yet increased even after complete sugars exhaustion to reach 35.29 g L^−1^ at day 10 (Fig. 2). Jin et al. [37] also observed that monocarbohydrates were initially accumulated before being taken up for conversion into mycelial biomass (from 7.5 to 9.2 g L^−1^) during aerobic fermentation of a raw starch processing wastewater, with either *Aspergillus oryzae* or *Rhizopus oligosporus*. This accumulation is most likely occurring when the rate of complex polymers hydrolysis is higher than the rate of carbohydrate uptake for cell growth.

When looking more carefully at the biomass production profile (Fig. 2), initial growth occurring during the first 120 h (day 5) did not appear to occur exponentially, but rather linearly. This observation would strengthen the hypothesis that the growth is mainly limited by the availability of fermentable sugars which are slowly and linearly produced through the activity of specific hydrolases from *A. niger* acting on complex polymers.

#### Organic acids utilization

It is known that SDW naturally contains large amount of organic acids [5]. Some of them were assayed in raw SDW filtrate and concentration of 5.7 ± 0.51 g L^−1^ for trans-aconitic acid, 2.8 ± 0.76 g L^−1^ for citric acid, 2.5 ± 0.47 g L^−1^ for isocitric acid, 0.7 ± 0.25 g L^−1^ for itaconic acid and 0.6 ± 0.18 g L^−1^ for oxalic acid were measured (Table 2). When sugars are being consumed by the cells, the concentration of itaconic, isocitric and oxalic acids tended to increase in the culture medium (+ 26, + 12 and + 136% respectively). An inverse relationship between consumption of sugars and organic acid production was already observed by [38] who reported that the maximum acid production was found for 6 days old *A. niger* cultures. Opposite tendency was noticed for citric and trans-aconitic acids that were slightly consumed during the first 7 days of growth. However, except for itaconic acid for which concentration remained stable, all organic acids were consumed partially or completely in the remaining 3 days (Table 2). These data indicated that organic acids were preferentially consumed during the second period of fermentation after complete exhaustion of monosaccharides. These carbon sources could be the result of hydrolysis of melanoidins, polyphenols or proteins present in crude SDW [39].

**Table 2:** Concentration (g L^−1^) of organic acids and pH measured in SDW filtrates after 0, 7 and 10 days of aerobic fermentation by *A. niger*

Organic acids	Concentrations			
	Day 0	Day 5	Day 7	Day 10
Itaconic acid	0.70 ± 0.25	0.63 ± 0.26	0.88 ± 0.22	0.87 ± 0.28
Trans-aconitic acid	5.71 ± 0.51	4.54 ± 1.4	4.32 ± 0.77	1.59 ± 0.37
Citric acid	2.84 ± 0.76	3.37 ± 0.28	1.36 ± 0.91	Bd
Isocitric acid	2.47 ± 0.46	2.52 ± 0.17	2.77 ± 0.41	Bd
Oxalic acid	0.61 ± 0.18	0.52 ± 0.14	1.45 ± 0.53	0.38 ± 0.14
pH	4.6 ± 0.1	5.07 ± 0.49	5.93 ± 1.21	5.37 ± 0.13

Each value is a mean of at least three independent experiments

*Bd* below detection level

Taken together, these results showed that growth of *A. niger* on SDW is a complex process. Free carbohydrates initially present in the media (namely glucose, fructose and mannitol) and other fermentable sugars probably released from complex polymers through hydrolytic activity of the fungal enzymes, are first consumed during the early growth phase. When free sugars disappeared from the medium (after 7–8 days of culture), growth continued on the free organic acids accumulated in the medium as well as other sugars released by *A. niger* hydrolases.

### SDW biochemical fingerprints

#### Global EEM profiles of raw and pre-treated SDW

**Fig. 3:**
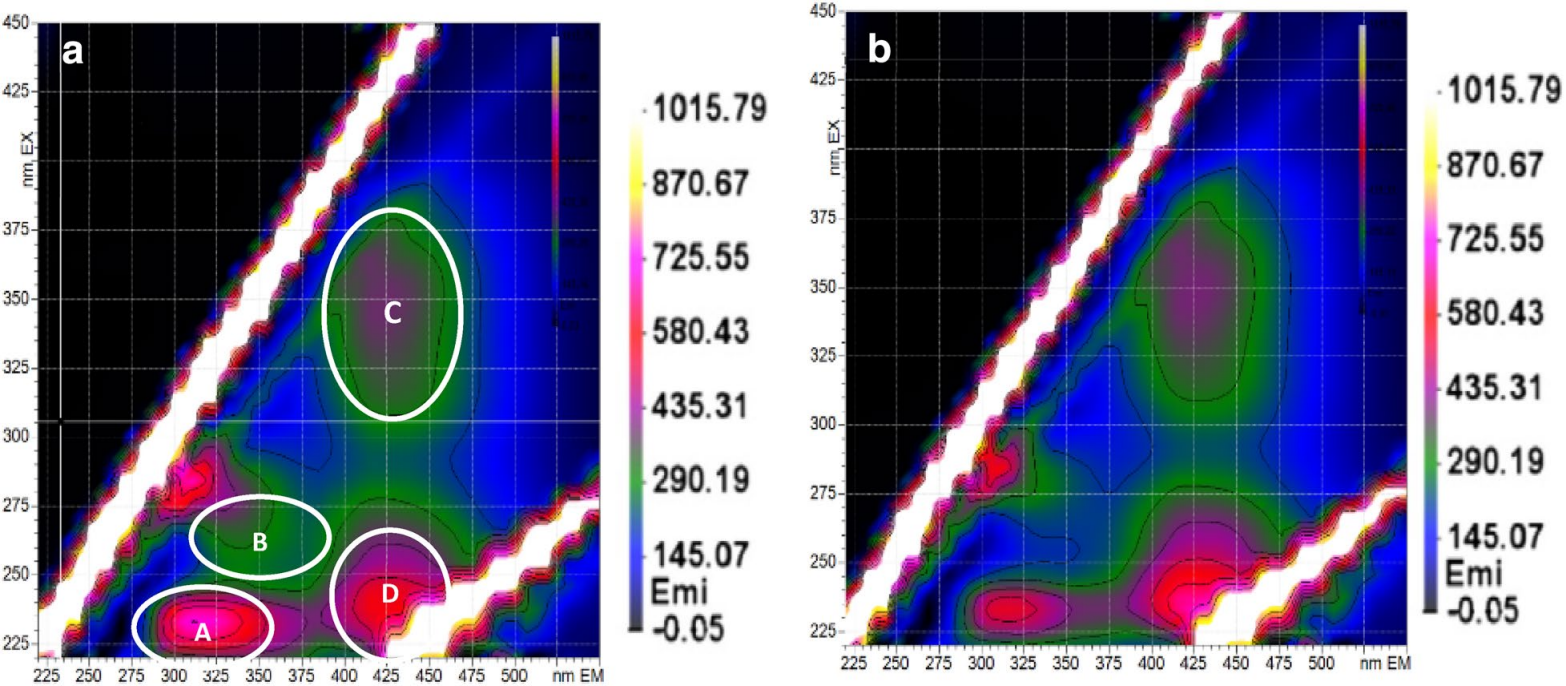
Analysis of the fluorescent matter in raw SDW filtrate (**a**) and in SDW filtrate treated for 5 days with *A. niger* (**b**) according to classification provided for wastewater: peak (A) corresponds to protein-like (PN-like) substances [40] and peak (B) to phenolic acid-like (PA-like) compounds [41]; peaks (C) and (D) can be related to humic acid-like (HA-like) substances [42]

Three-dimensional excitation emission matrixes (EEM) were determined on the SDW filtrates in order to detect potential modification of complex dissolved organic matter like melanoidins or phenolic acids during *A. niger* fermentation (Fig. 3). EEM is a widely used non-degradative method for qualitative characterization of the soluble substances of many effluents [43]. As proposed by [40], for typical wastewater spectra, the EEM can be divided in at least two regions: (1) the region with emission wavelength λ_Em_ < 380 nm which is associated with fluorescent molecules types A and B containing a limited number of aromatic rings like phenols, indole moiety and free tryptophan; and (2) the region with λ_Em_ > 380 nm which is associated with polycyclic aromatic fluorophores (types C and D) such as Humic acid, flavonoid and quinone. In addition, EEM of pure gallic acid was performed to locate more precisely its associated zone: EEM showed a fluorescent peak (250 < λ_Ex_ < 275 nm and 325 < λ_Em_ < 370 nm) that was included in the phenolic acid-like (PA-like) region (type B). The determination of PA-like area zone in SDW is in accordance with [44] who worked on partially degraded food waste and undigested dietary fibres.

Concerning the SDW media, both matrixes were composed by four peaks with similar excitation/emission wavelengths (λ_Ex_/λ_Em_) position and intensity (Fig. 3a, b): (1) for λ_Em_ < 380 nm, peak A and peak B were located in the regions corresponding to protein-like (PN-like) and phenolic acid-like (PA-like) compounds respectively [41] and peak A was much more intense than peak B (2) for λ_Em_ > 380 nm, peaks D and C were associated with quinine-like components and could be related to humic acid-like (HA-like) substances [42]. These results were in good accordance with the results obtained by [45] which highlighted these groups of fluorophores (A, B and C-D areas) in sugarcane vinasse. In this way, EEM determined on the soluble SDW fractions (treated or not) did not allow to clearly show EEM modifications pattern related to *A. niger* metabolism (four independent replicates were analysed; only one replicate was shown). This can be explained by the complexity of the SDW medium that contains highly fluorescent molecules possibly covering the detection of other ones. Moreover, only specific molecules with aromatic ring are detected by EEM.

#### EEM profiles after size fractionation of raw and fermented SDW

In order to evaluate whether the SDW has been altered by *A. niger* treatment, size fractionation of raw and 5-days fermented SDW was chosen to provide a synthetic view of their composition and size distribution. SEC chromatograms were first monitored by absorbance detection at 210 nm and 280 nm but raw and fermented SDW filtrates displayed similar profiles (data not shown). Regarding the EEM spectra of the two SDW samples (Fig. 3a, b), high fluorescence intensities could be noticed on the PN-like region (peak A). One common couple of wavelengths (λ_Ex_/λ_Em_ = 221/350 nm) that was previously described by [43] for detection of tryptophan containing PN-like molecules was then selected for SEC monitoring. Fluorescent compounds detected in this region (peak A) were reported by [46] as soluble microbial products associated to microbial activity or to cellular material.

**Fig. 4:**
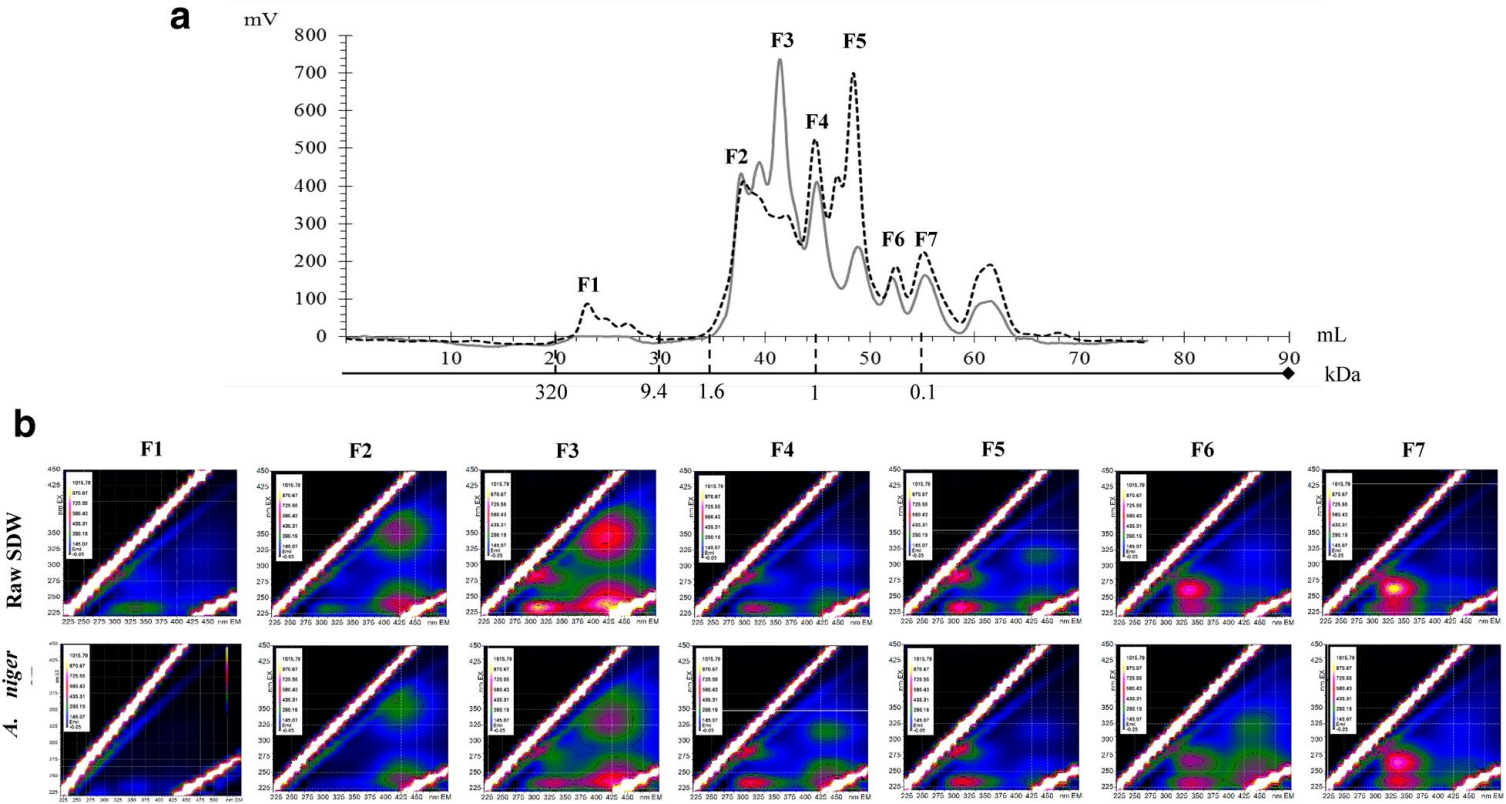
Size exclusion profiles of raw SDW (bold lines) or SDW treated for 5 days with *A.* *niger* (dotted lines) monitored at the λ_Ex_/λ_Em_ = 221 nm/350 nm and corresponding to the injection of 1 mg of soluble COD (**a**). Seven fractions (F1 to F7) were collected and performed through EEM fluorescence at λ_Ex_ comprised between 220 and 450 nm and λ_Em_ from 220 to 500 nm (**b**)

Fractionation of the raw and fermented SDW filtrates was performed by SEC and could be divided in seven fractions from F1 to F7 corresponding to increasing elution volume and to decreasing apparent molecular size (Fig. 4a). Quantitative repartition of each fraction among all the eluted molecules was also evaluated after peak area integration (Table 3). It can be noticed that fermented SDW showed some early eluted molecules in the F1 fraction that were not present in raw SDW. According to the calibration curve (see “EEM profiles obtained from filtrates of crude and pre-treated SDW” section), these PN-like molecules that eluted around 24 mL had high apparent molecular weight around 100,000 Da. Also, molecules with very small molecular weight, eluted in the F6 and F7 fractions, were found in both profiles with a similar repartition. The PN-like molecules included in the F2 fraction (around 1000 Da) were not fully digested during *A. niger* fermentation since they still represent 25.7% of the total molecules (Table 3). On the other hand, a drastic diminution of F3 peak, and an increase of the F5 peak were observed in fermented compared to raw SDW filtrate (Fig. 4a, Table 3). It is possible that the decrease of F3 from 31.6% in the untreated SDW to 8.73% after *A. niger* is recovered in the F5 peak that has increased from 7.7 to 18.63% of the total SEC area. These observations suggest that molecules with intermediate size (F3) might have been partially hydrolysed in small molecules (F5) after 5 days of *A. niger* fermentation in SDW. This is in agreement with the fact that after a first growth period of *A. niger* on released monosaccharides, other complex polymeric molecules need to be hydrolysed to provide additional carbon sources. The high apparent molecular weight molecules detected in the fermented SDW (F1) might thus correspond to enzymes secreted by the biomass for hydrolysis of SDW carbon-containing polymers.

**Table 3:** Biochemical properties of the fractions eluted after SEC fractionation of raw and fermented SDW: quantitative distribution of each fraction and ratio of maxima fluorescence intensity for the three A, B and C peak areas detected in their EEM

Fractions	Peak (mL)	Repartition of peak area compared to total area (%)		A/B EEM peak area		A/C EEM peak area	
		Raw SDW	Fermented SDW	Raw SDW	Fermented SDW	Raw SDW	Fermented SDW
F1	24 ± 2	–	5.31	–	2.0	–	3.9
F2	38 ± 1	12.1	25.67	1.9	1.8	0.6	0.4
F3	41.5 ± 1.5	31.6	8.73	1.8	1.8	1.2	3.3
F4	45 ± 1	15.3	14.32	2.2	2.2	2.5	2.2
F5	49 ± 1.5	7.7	18.63	1.7	1.7	2.6	4.4
F6	52.25 ± 1.25	2.7	4.02	0.7	1.1	3.5	1.8
F7	55.75 ± 2.25	7.1	7.77	0.6	0.8	5.1	4.0

A corresponds to the maximum intensity of the peak area related to protein-like (PN-like) substances, B to phenolic gallic acid-like (PA-like) molecules and C to the humic acid-like (HA-like) substances. SEC and EEM profiles were obtained from supernatants of SDW pre-treated by *A. niger* during 5 days

To further investigate the effect of *A. niger* fermentation on the biochemical characteristics of vinasse, F1 to F7 fractions were collected and their EEM were determined (Fig. 4b). For better specificity towards PN-like detection, the ratios A/B and A/C of maxima fluorescence intensity for these different zones were calculated (Table 3). As shown in Fig. 4b, EEM fingerprints were quite similar for raw and fermented SDW. Globally, F2 fraction was more enriched in HA-like substances (ratio peak C/A more important for F2 fraction than for others) whereas F7 fraction was especially enriched in PA-like molecules (ratio peak B/A more important for F7 fraction and especially for SDW filtrate). EEM fingerprints of F3, F5 and F6 fractions were slightly impacted by fermentation. For F3 and F5, the A/C ratio was increased by a factor of 2.8 and 1.7 after fermentation respectively whereas A/B ratio was unchanged. That might be linked to the increase in PN-like and/or the hydrolysis of HA-like molecules during *A.* *niger* fermentation. On the other hand, concerning F6 fractions, the A/C ratio was reduced by a factor 1.9, decreasing from a value of 3.5–1.8 after fermentation. So, fermentation has decreased the level of HA-like substances in fractions F3 and F5 whereas these substances were recovered in a higher amount in F6 fractions.

According to these results, some physiological aspects of *A. niger* fermentation of raw SDW can be proposed: (1) production of high apparent molecular weight (F1 fraction) and hydrolysed (F3 and F5 fractions) PN-like molecules (2) hydrolysis of HA-like substances (F3 and F5 fractions) in smaller HA-like molecules, (F6 fraction) inducing vinasse decolourisation. This approach also demonstrated that SEC coupled with fluorescence monitoring at λ_Ex_/λ_Em_ = 221/350 nm is a good alternative for determination of vinasse biochemical fingerprints. This strategy was previously used to show the impact of biological aggregate sludge and origin of aggregate on exopolymeric substances fingerprint for which number of peaks and their intensity were easily identified with the specific PN-like fluorescence detection [43].

### Lipid extraction from *A. niger* biomass and total Single Cell Oil yield

In an attempt to explore the potential of fungal biomass for biodiesel production, the lipid content of the biomass produced on SDW was measured and compared with the one produced on a lipid accumulation medium (Table 4). With almost 3 times more biomass produced on SDW as compared to LAM, the lipid content of the fungal biomass grown on SDW (6.94%) was slightly higher as compared to LAM (5.89%). Comparatively, Zheng et al. [47] showed that *A. niger* grown on glucose or xylose as sole carbon source led to a production of 5.8 and 4.6 g L^−1^ of biomass with a lipid content of 9.6 and 8% respectively. Similarly, *A. niger* grown on bagasse led to a fungal biomass of about 1.9 g L^−1^ with a lipid content of 13.6% [48]. Also, André et al. [49] showed that two different *A.* *niger* strains cultivated in crude glycerol could produce up to 8.2 g L^−1^ of biomass with a lipid content of about 50% (meaning about 3 g L^−1^ of lipids). Although lipid content of fungal biomass produced on SDW is rather low (circa 7%), the high *A.* *niger* biomass yield on this medium suggested that SDW can therefore constitute a good alternative and cheap medium for biodiesel production.

**Table 4:** Biomass production, lipid content and lipid composition of *A. niger* grown on LAM and SDW media during 10 days

Medium	Biomass	Lipid content^a^	Lipid composition				
	(g L^−1^)	(% of DW)	16:0	18:0	18:1 (n-9)	18:2 (n-6)	18:3 (n-3)
LAM	8.523	5.889	18.19	6.84	28.19	39.38	7.4
SDW	24.060	6.940	24.94	5.25	17.23	42.66	9.92

16:0: palmitic acid; 18:0: stearic acid; 18:1 (n-9): oleic acid; 18:2 (n-6): linoleic acid; 18:3 (n-3): linolenic acid

^a^Lipid content expressed in gram of lipids per 100 g of dry weight biomass

The composition of the lipids extracted from biomass produced on LAM and SDW was respectively 18.2 and 24.9% for palmitic acid (16:0), 28.2 and 17.2% for oleic acid (18:1, n-9) and 39.4 and 42.7% for linoleic acid (18:2, n-6). Stearic (18:0) and α-linolenic (18:3, n-3) acids were produced to a lesser extent by *A.* *niger* on both media (Table 4). Singh [48] reported that *A.* *niger* biomass grown on glucose medium contained mostly linoleic acid (50%) and to a lesser extent, palmitic, stearic and linolenic acids (8.3, 5.2 and 6% respectively). Whatever the medium used, linolenic acid appeared as the major intracellular lipid of *A.* *niger* biomass; however, it can be noticed that *A.* *niger* grown on glucose medium and on LAM were richer in oleic acid than biomass grown on SDW (23.5 and 28.19 against 17.23%) [48]. By comparison, biodiesel from *Yarrowia lipolytica* [50] contained twice higher oleate esters but less than three times linoleate esters than biodiesel from *A.* *niger* grown on SDW. This suggests that lipids produced from *A.* *niger* could be an interesting alternative to the ones produced by microorganisms such as yeasts [51].

Finally, the main relevant physical properties to assess fuel quality of biodiesel from *A. niger* are presented in Table 5. Whatever the growth media used (SDW or LAM), the biodiesel derived from *A.* *niger* showed similar properties for all the tested physical parameters such as cetane number (ϕ), viscosity (η), density (*ρ*), higher heating value (HHV—δ) and cold filter plugging point (CFPP). Cetane numbers of biodiesel produced from SDW and LAM media (respectively 57.65 and 58.88) were both at least 13% better than the minimal requirement of biodiesel proposed by the European and American standards. For comparison, the biodiesel derived from *A.* *niger* had similar cetane numbers to biodiesels produced from coconut, tallow or yellow grease (respectively 59.3, 58.9 and 56.9) [51]. In comparison, cetane number of biodiesel from *Y. lipolityca* was 64.37 [50]. Also viscosities of biodiesel produced from *A. niger* (3.52 mm^2^ s^−1^ on LAM and 3.47 mm^2^ s^−1^ on SDW) were globally in the range of values suggested by the European Standards (between 3.5 and 5 mm^2^ s^−1^). Although there are no European or American specification for this parameter, HHV of biodiesel from *A. niger* grown on SDW (40.01 MJ kg^−1^) is considered as acceptable given that biodiesel from all kind of sources are generally 10% less energetic than diesel from petroleum (49.65 MJ kg^−1^) [51]. Finally, CFPP value of biodiesel obtained from *A. niger* grown on SDW was lower than 0 °C, meaning that this biodiesel could be used at low temperature.

**Table 5:** Most relevant physical characteristics of biodiesel extracted and converted from *A. niger* biomass grown on LAM and SDW media during 10 days

	CN (ϕ)	Viscosity (η) (mm^2^ s^−1^)	Density (*ρ*) (g cm^−3^)	HHV (δ) (MJ kg^−1^)	CFPP (°C)
SDW	57.65	3.47	0.87	40.01	− 0.39
LAM	58.88	3.52	0.87	40.05	− 0.02
EN 14,214^a^	> 51^b^	3.5–5^c^	0.86–0.9^b^	–	< − 15^d,1^; < 0^d,2^
ASTM D 6751-08^a^	> 47^b^	1.9–6^c^	n.a	–	n.a

Each data is the mean of three independent biological experiments

*CN* cetane number, *HHV* higher heating value, *CFPP* cold filter plugging point (^1^in winter, ^2^in summer), *n.a* not available

^a^According to European and American specifications biodiesel fuel blendstocks (B100), standard specifications EN 14,214 and D 6751-08 for biodiesel fuel blendstocks (B100) established respectively by the European Committee for Standardization (CEN) and American Society for Testing and Materials (ASTM). Data from ^b^ [52], ^c^ [53], ^d^ [54]

## Conclusions

This study demonstrated that raw SDW contains suitable organic substrates for growth of *A. niger* including monosaccharides, organic acids and complex polymers. The growth reached up to 35.29 g L^−1^ DW fungal biomass with a biomass yield of 0.47 g per g of SDW organic compounds. Aerobic fermentation of raw SDW led to vinasse decolourization with pH increase and COD decrease, dropping thus significantly the pollutant load. Biochemical fingerprints revealed that high molecular weight PN-like components were secreted by *A. niger* during growth while some PA and/or HA-like molecules were consumed. Intracellular lipids from biomass showed good physical characteristics for use as biofuel giving new insights for concomitant bioremediation and carbon reuse of SDW medium.

## Additional file

**Additional file 1.** Data from Fig. 2 including standard deviations.

## References

[1] Lima AM, Souza RRD (2014). Use of sugar cane vinasse as substrate for biosurfactant production using Bacillus subtilis PC. Chem Eng Trans.

[2] Bhattacharyya A, Pramanik A, Maji S, Haldar S, Mukhopadhyay U, Mukherjee J (2012). Utilization of vinasse for production of poly-3-(hydroxybutyrate-co-hydroxyvalerate) by *Haloferax mediterranei*. AMB Express.

[3] Tewari PK, Batra VS, Balakrishnan M (2007). Water management initiatives in sugarcane molasses based distilleries in India. Resour Conserv Recycl.

[4] Wilkie AC, Riedesel KJ, Owens JM (2000). Stillage characterization and anaerobic treatment of ethanol stillage from conventional and cellulosic feedstocks. Biomass Bioenergy.

[5] Fuess LT, Garcia ML (2014). Implications of stillage land disposal: a critical review on the impacts of fertigation. J Environ Manag.

[6] Baez-Smith C. Anaerobic digestion of vinasse for the production of methane in the sugar cane distillery. In: SPRI Conference on Sugar Processing Research, Águas de São Pedro, S.P., Brazil. 2006.

[7] Rajagopal V, Paramjit SM, Suresh KP, Yogeswar S, Nageshwar RDVK, Avinash N (2014). Significance of vinasses waste management in agriculture and environmental quality—review. Afr J Agric Res.

[8] Acharya BK, Mohana S, Madamwar D (2008). Anaerobic treatment of distillery spent wash—a study on upflow anaerobic fixed film bioreactor. Bioresour Technol.

[9] España-Gamboa E, Mijangos-Cortes J, Barahona-Perez L, Dominguez-Maldonado J, Hernández-Zarate G, Alzate-Gaviria L (2011). Vinasses: characterization and treatments. Waste Manag Res.

[10] Mohana S, Acharya BK, Madamwar D (2009). Distillery spent wash: treatment technologies and potential applications. J Hazard Mater.

[11] Bustamante MA, Paredes C, Moral R, Moreno-Caselles J, Pérez-Espinosa A, Pérez-Murcia MD (2005). Uses of winery and distillery effluents in agriculture: characterization of nutrient and hazardous components. Water Sci Technol.

[12] Khairnar P, Chavan F, Diware VR (2013). Generation of energy from distillery waste water. Int J Sci Spiritual Bus Technol.

[13] Biswas AK, Mohanty M, Hati KM, Misra AK (2009). Distillery effluents effect on soil organic carbon and aggregate stability of a Vertisol in India. Soil Tillage Res.

[14] Ansari F (2014). Environmental impact of distillery effluent on vertical soil horizon due to leaching effect: an experimental approach. Int J Chem Environ Eng.

[15] Pant D, Adholeya A (2007). Biological approaches for treatment of distillery wastewater: a review. Bioresour Technol.

[16] Kanimozhi R, Vasudevan N (2010). An overview of wastewater treatment in distillery industry. Int J Environ Eng.

[17] Palacios-Cabrera H, Taniwaki MH, Hashimoto JM, Menezes HC (2005). Growth of *Aspergillus ochraceus*, *A. carbonarius* and *A. niger* on culture media at different water activities and temperatures. Braz J Microbiol.

[18] Schrickx JM, Raedts MJH, Stouthamer AH, Vanverseveld HW (1995). Organic acid production by *Aspergillus niger* in recycling culture analyzed by capillary electrophoresis. Anal Biochem.

[19] Quintanilla D, Hagemann T, Hansen K, Gernaey KV (2015). Fungal morphology in industrial enzyme production—modelling and monitoring. Adv Biochem Eng Biotechnol.

[20] Schuster E, Dunn-Coleman N, Frisvad JC, Van Dijck PWM (2002). On the safety of *Aspergillus niger*—a review. Appl Microbiol Biotechnol.

[21] Oshoma CE, Imarhiagbe EE, Ikenebomeh MJ, Eigbaredon HE (2010). Nitrogen supplements effect on amylase production by *Aspergillus niger* using cassava whey medium. Afr J Biotechnol.

[22] Rosalem P, Tauk S, Santos MCN (1985). Efeito da temperatura, pH, tempo de cultivo e nutrientes no crescimento de fungos imperfeitos em vinhaca. Rev microbiologia.

[23] Silveira Ruegger MJ, Tauk-Tornisielo SM (1996). Biomass production by filamentous fungi in sugar cane vinasse medium supplemented with molasses. Arq Biol Tecnol.

[24] Sluiter A, Hames B, Scarlata C, Sluiter J, Templeton D. Determination of ash in biomass (No. NREL/TP-510-42622). National Renewable Energy Laboratory of U.S. Department of Energy, Golden, US. 2008.

[25] Janke L, Leite A, Nikolausz M, Schmidt T, Liebetrau J, Nelles M, Stinner W (2015). Biogas production from sugarcane waste: assessment on kinetic challenges for process designing. Int J Mol Sci.

[26] Suutari M, Priha P, Laakso S (1993). Temperature shifts in regulation of lipids accumulated by *Lipomyces starkeyi*. J Am Oil Chem Soc.

[27] Simon S, Païro B, Villain M, D’Abzac P, Van Hullebusch E, Lens P, Guibaud G (2009). Evaluation of size exclusion chromatography (SEC) for the characterization of extracellular polymeric substances (EPS) in anaerobic granular sludges. Bioresour Technol.

[28] Cropotova J, Popel S, Parshacova L, Colesnicenco A (2015). Effect of 1-year storage time on total polyphenols and antioxidant activity of apple fillings. J Food Packag Sci Tech Technol.

[29] Hoarau J, Caro Y, Petit T, Grondin I (2016). Evaluation of direct wet transesterification methods on yeast and fungal biomass grown on sugarcane distillery spent wash. Chem Eng Process Technol.

[30] Sangave PC, Pandit AB (2006). Enhancement in biodegradability of distillery wastewater using enzymatic pretreatment. J Environ Manag.

[31] España-Gamboa EI, Mijangos-Cortés JO, Hernández-Zárate G, Maldonado JAD, Alzate-Gaviria LM (2012). Methane production by treating vinasses from hydrous ethanol using a modified UASB reactor. Biotechnol Biofuels.

[32] Ferreira LFR, Aguiar MM, Messias TG, Pompeu GB, Lopez AMQ, Silva DP, Monteiro RT (2011). Evaluation of sugarcane vinasse treated with *Pleurotus sajor*-*caju* utilizing aquatic organisms as toxicological indicators. Ecotoxicol Environ Saf.

[33] Sheehan GJ, Greenfield PF (1980). Utilization, treatment and disposal of distillery wastewater. Water Res.

[34] Miranda MP, Benito GG, Cristobal NS, Nieto CH (1996). Color elimination from molasses wastewater by *Aspergillus niger*. Bioresour Technol.

[35] Patil PU, Kapadnis BP, Dhamankar VS (2003). Decolorisation of synthetic melanoidin and biogas effluent by immobilised fungal isolate of *Aspergillus niger* UM2. Int Sugar J.

[36] Cavka A, Jönsson LJ (2014). Comparison of the growth of filamentous fungi and yeasts in lignocellulose-derived media. Biocatal Agric Biotechnol.

[37] Jin B, Yan XQ, Yu Q, van Leeuwen JH (2002). A comprehensive pilot plant system for fungal biomass protein production and wastewater reclamation. Adv Environ Res.

[38] Khosravi-Darani K, Zoghi A (2008). Comparison of pretreatment strategies of sugarcane baggase: experimental design for citric acid production. Bioresour Technol.

[39] Agarwal R, Lata S, Gupta M, Singh P (2010). Removal of melanoidin present in distillery effluent as a major colorant: a review. J Environ Biol (India).

[40] Li WT, Chen SY, Xu ZX, Li Y, Shuang CD, Li AM (2014). Characterization of dissolved organic matter in municipal wastewater using fluorescence PARAFAC analysis and chromatography multi-excitation/emission scan: a comparative study. Environ Sci Technol.

[41] Lakowicz JR (2006). Principles of fluorescence spectroscopy.

[42] Huang M, Li Y, Gu G (2010). Chemical composition of organic matters in domestic wastewater. Desalination.

[43] Bhatia D, Bourven I, Simon S, Bordas F, van Hullebusch ED, Rossano S, Lens PNL, Guibaud G (2013). Fluorescence detection to determine proteins and humic-like substances fingerprints of exopolymeric substances from biological sludges performed by size exclusion chromatography. Bioresour Technol.

[44] Pokhrel D, Viraraghavan T (2004). Treatment of pulp and paper mill wastewater—a review. Sci Total Environ.

[45] Soobadar A. Agronomic and environmental impacts of application of coal/bagasse ash and vinasse to sugarcane fields in Mauritius (PhD thesis). Université d’Avignon, Avignon, France. 2009.

[46] Bridgeman J, Baker A, Carliell-Marquet C, Carstea E (2013). Determination of changes in wastewater quality through a treatment works using fluorescence spectroscopy. Environ Technol.

[47] Zheng Y, Yu X, Zeng J, Chen S (2012). Feasibility of filamentous fungi for biofuel production using hydrolysate from dilute sulfuric acid pretreatment of wheat straw. Biotechnol Biofuels.

[48] Singh A (1992). Lipid accumulation by a cellulolytic strain of *Aspergillus niger*. Experientia.

[49] André A, Diamantopoulou P, Philippoussis A, Sarris D, Komaitis M, Papanikolaou S (2010). Biotechnological conversions of bio-diesel derived waste glycerol into added-value compounds by higher fungi: production of biomass, single cell oil and oxalic acid. Ind Crops Prod.

[50] Katre G, Joshi C, Khot M, Zinjarde S, RaviKumar A (2012). Evaluation of single cell oil (SCO) from a tropical marine yeast *Yarrowia lipolytica* NCIM 3589 as a potential feedstock for biodiesel. AMB Express.

[51] Hoekman SK, Broch A, Robbins C, Ceniceros E, Natarajan M (2012). Review of biodiesel composition, properties, and specifications. Renew Sustain Energy Rev.

[52] Ramírez-Verduzco LF, Rodríguez-Rodríguez JE, del Rayo Jaramillo-Jacob A (2012). Predicting cetane number, kinematic viscosity, density and higher heating value of biodiesel from its fatty acid methyl ester composition. Fuel.

[53] Ramos MJ, Fernández CM, Casas A, Rodríguez L, Pérez Á (2009). Influence of fatty acid composition of raw materials on biodiesel properties. Bioresour Technol.

[54] Su YC, Liu YA, Diaz-Tovar CA, Gani R. Selection of prediction methods for thermophysical properties for process modeling and product design of biodiesel manufacturing (thesis). Virginia Tech. 2011.

