# Supplementary figures and images for: Production of Aspergillus niger biomass on sugarcane distillery wastewater: physiological aspects and potential for biodiesel production
Source: Fungal Biol Biotechnol. 2018 Jan 16;5:1. doi: 10.1186/s40694-018-0045-6 (PMC5771024; doi:10.1186/s40694-018-0045-6)

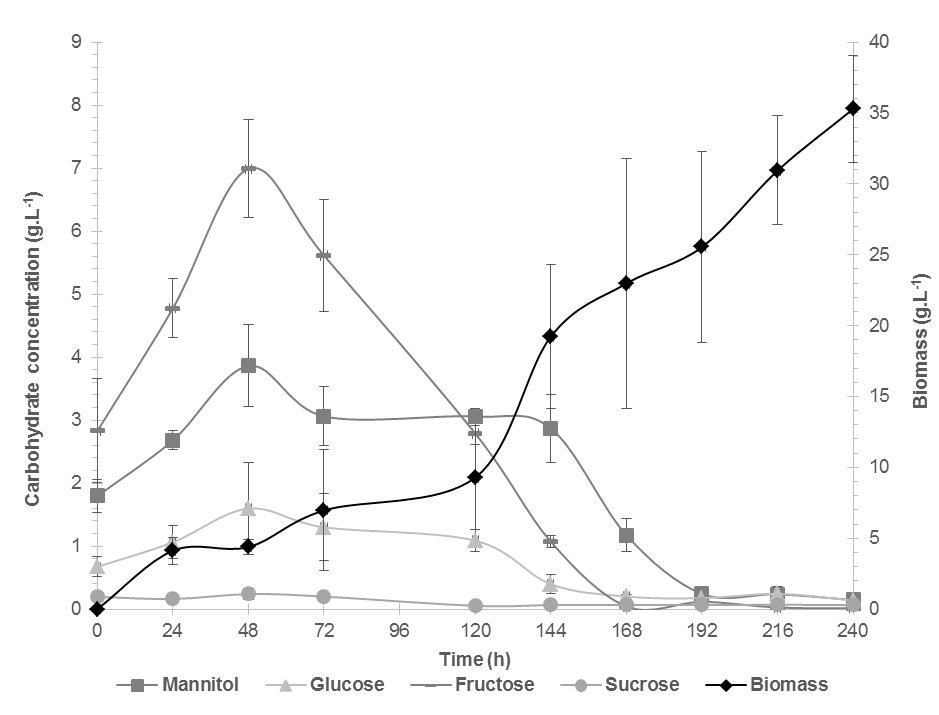

Supplement: Supplementary file 1 — Additional file 1. Data from Fig. 2 including standard deviations. [file 40694_2018_45_MOESM1_ESM.docx]
